# Clinical Results of Acetabular Fracture *via* the Pararectus *versus* Ilioinguinal Approach

**DOI:** 10.1111/os.12970

**Published:** 2021-05-04

**Authors:** Ruyi Zou, Min Wu, Jianzhong Guan, Yuzhou Xiao, Xiaotian Chen

**Affiliations:** ^1^ Department of Orthopaedics The First Affiliated Hospital of Bengbu Medical College Bengbu China

**Keywords:** Acetabular fracture, Fracture fixation, Ilioinguinal approach, Pararectus approach, Surgical method

## Abstract

**Objective:**

To compare the clinical efficacy of pararectus and ilioinguinal approach in the treatment of acetabular fractures.

**Methods:**

A retrospective analysis of the clinical data of 60 patients with acetabular fractures treated by the pararectus approach or the ilioinguinal approach from January 2016 to January 2019 was performed to record all data by comparing the length of the surgical incision, the time to expose the fracture and the amount of blood loss during the operation. Patients were routinely followed up at 1, 6 and 12 months postoperatively. The function of the hip joint after the operation (Improved Merle d' Aubigne and Postel scores) and the complications postoperation were recorded.

**Results:**

There was a significant difference (mean ± SD) in the length of surgical incision [(11.2 ± 1.5) cm *vs*.(23.8 ± 2.1) cm], and in surgical exposure time [(10.8 ± 1.7) min *vs*.(19.9 ± 1.9) min] (*P* < 0.05) between the two approaches; there was no significant difference (mean ± SD) in intraoperative blood loss [(591.8 ± 131.4) mL *vs*. (614.6 ± 132.7) mL] or in hip function scores at the last follow‐up between the two groups (*P* > 0.05). In the pararectus approach group, there was one patient (3.3%) with postoperative wound fat liquefaction, and the wound completely improved by secretion culture, enhanced dressing and effective antibiotics, one patient (3.3%) developed lateral femoral cutaneous nerve injury; One case (3.3%) of postoperative myositis ossificans occurred in the ilioinguinal approach group, and there were no obvious symptoms.

**Conclusions:**

These data suggest that for patients with acetabular fractures, both the pararectus approach and the ilioinguinal approach can achieve satisfactory surgical results, but the former has relatively simple operation and small incision length, which is in line with the modern concept of the minimally invasive pelvis.

## Graphical abstract

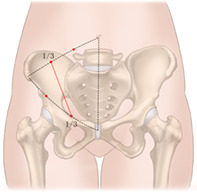

## Introduction

Acetabular fractures are usually associated with some high‐energy injuries. Due to its exceptional location, open reduction and internal fixation have become the “gold standard” for the treatment of displaced acetabular fractures^1^, ^2^, ^3^. For the surgical method of anterior acetabular fracture, more reports are the pararectus approach^4^, the ilioinguinal approach^5^ and the modified Stoppa approach^6^.

In the classic work of Judet and Letournel, the ilioinguinal approach was described in detail. This approach can fully expose the anterior column, the surface of the square area and the upper and lower branches of the pubic bone, while not interfering with the hip abductors, which is conducive to the early rehabilitation of patients. Owing to this surgical approach can fully expose the front of the acetabulum which has always been a classic approach to the treatment of acetabular anterior fractures^5^. However, the incision of this approach is long and requires three windows to expose. If there is a separation of the pubic symphysis, four windows are required to expose. The surgical exposure takes a long time and it is difficult to shape the plate during the operation. At the same time, due to the complicated local anatomy, it is easy to merge with vascular and nerve damage during the operation. The famous French hernia surgeon Stoppa created a method of inguinal hernia repair in 1969, that is, a huge patch to strengthen the visceral sac, also known as the Stoppa approach. In 1993, Hirvensalo *et al*.^6^ First applied the Stoppa approach to the treatment of pelvic fractures. They believed that compared with the traditional ilioinguinal approach, there was no need to expose the external iliac vascular bundle, iliopsoas muscle and femoral nerve, and the operation was simple and did not damage the lateral femoral cutaneous nerve. But, the Stoppa way still has certain limitations. For example, there is a specified distance from the iliac wing fracture. It is often necessary to use a tiny incision on the iliac crest to assist in the reduction. At the same time, because of the small incision, it is difficult to operate in patients with a severe displacement of the fracture, especially in obese patients. Professor Keel introduced a new surgical approach in 2012 and named it the pararectus approach^4^. The pararectus approach is based on the modified Stoppa approach. The surgical incision is moved to the side of the fracture and entered obliquely along the rectus abdominous in the Hesselbach triangle. Its deep surface faces the acetabulum, and the true pelvic rim can be revealed from the front and medial sides of the acetabulum. The exposure ranges from the pubic symphysis to the sacroiliac joints, including the square area of the acetabulum and the acetabulum inside the ischial body. He hopes that this approach can: (i) fully reveal the acetabular fracture; (ii)directly look at important structures such as blood vessels and nerves during the operation; and (iii) afford less soft tissue damage.

A retrospective analysis of the clinical data of 60 patients with acetabular fractures treated by the pararectus approach or the ilioinguinal approach from January 2016 to January 2019 was performed, the purpose of this study was to: (i) understand the main points during the operation of the two surgical approaches; (ii) know the precautions, indications, contraindications and complications of the two surgical approaches; and (iii)compare the clinical efficacy of the two surgical approaches and choose the appropriate surgical approach for patients with acetabular fractures.

## Materials and Methods

This study was approved by the local Ethics Committee of the Bengbu Medical College (Number: BYYFY‐2015KY23). All acetabular fractures that were operatively stabilized using either the pararectus or the ilioinguinal approach between January 2016 to January 2019. After admission to the hospital, patients with acetabular fractures should be given bone traction, the preoperative examination and condition assessment should be improved. Attention paid to observing whether there are combined injuries. If found, the relevant department in should be consulted. Two groups were created based on the surgical approach (A = pararectus and B = Ilioinguinal). There was no significant difference between the two groups in terms of gender ratio, age, cause of injury, and fracture type (Judet‐Letournal classification). For specific data refer to Table 1.

**TABLE 1:** Comparison of the general condition of the two groups of patients(^−^x ± s)

Group	(n)	Average age(year)	Gender(n)		Cause of injury(n)			Fracture classification(Judet‐Letournel)				
			Man	Woman	Car accident injury	Fall from height	Heavy injury	Anterior wall	Anterior column	Transverse	Double column	Anterior column with posterior semitransverse
A group	30	44.5 ± 16.6	18	12	20	6	4	4	3	3	15	5
B group	30	46.5 ± 16.7	19	11	18	6	6	3	2	5	13	7
Statistic	‐	*t* = −0.457	*χ* ^2^ = 0.071		*χ* ^2^ = 0.505			*χ* ^2^ = 1.467				
		*P* > 0.05	*P* > 0.05		*P* > 0.05			*P* > 0.05				

Group A is the pararectus approach and Group B is the ilioinguinal approach.

### Surgical Technique

#### Pararectus Approach

**Fig. 1:**
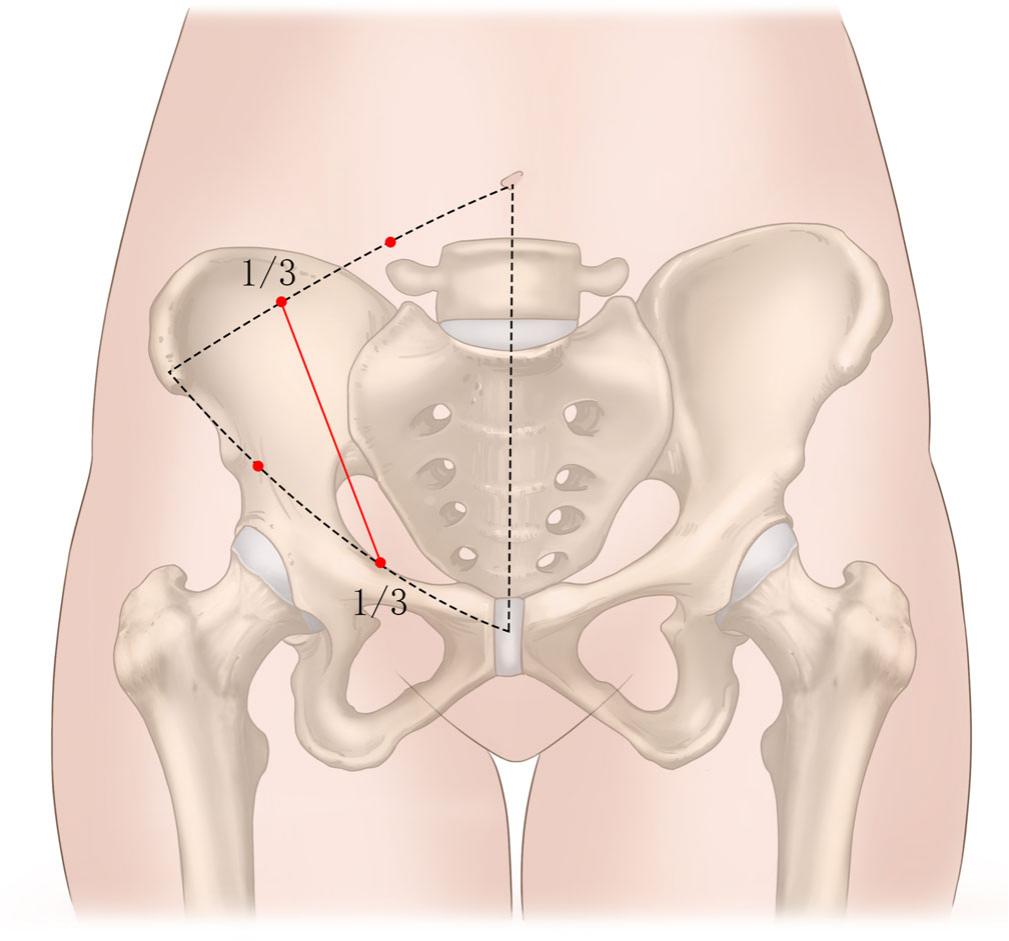
Landmarks and the skin incision of the pararectus approach. The incision starts at the middle and outer 1/3 of the line connecting the umbilical cord with the ASIS, and stops at the middle and inner 1/3 of the line connecting the ASIS with pubic symphysis.

**Fig. 2:**
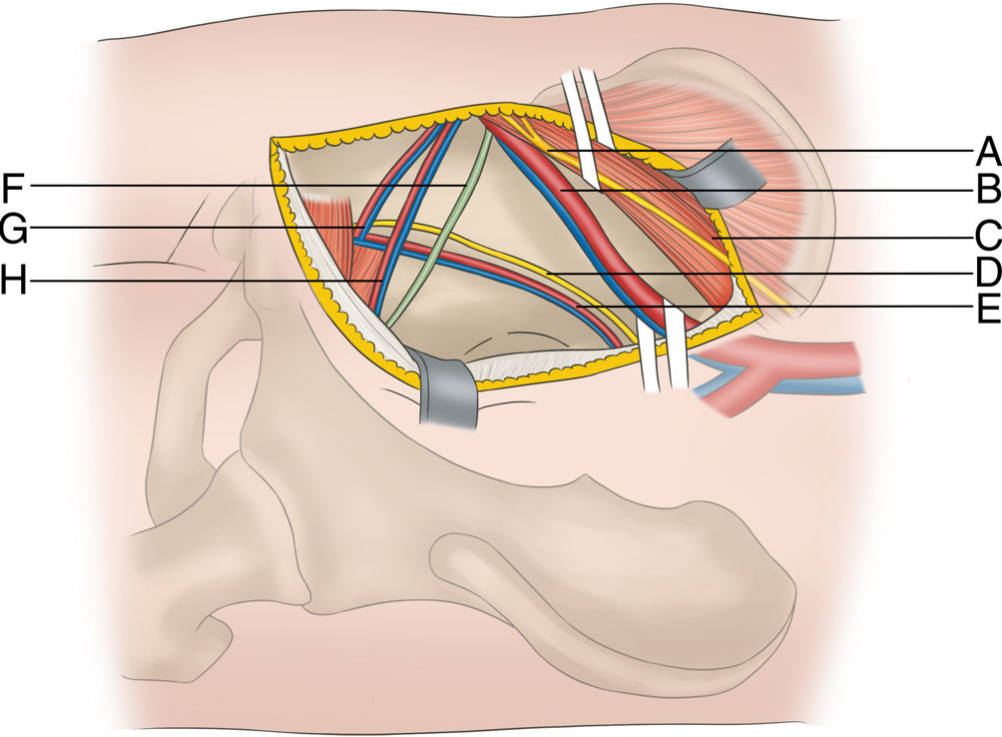
Diagram of the surgical exposure, showing (A) genital femoral nerve, (B) external iliac artery/vein, (C) iliac psoas muscle, (D) obturator nerve, (E) obturator vessels, (F) spermatic cord in men or round ligament in women, (G)”death crown”, (H) inferior abdominal artery/vein.

**Fig. 3:**
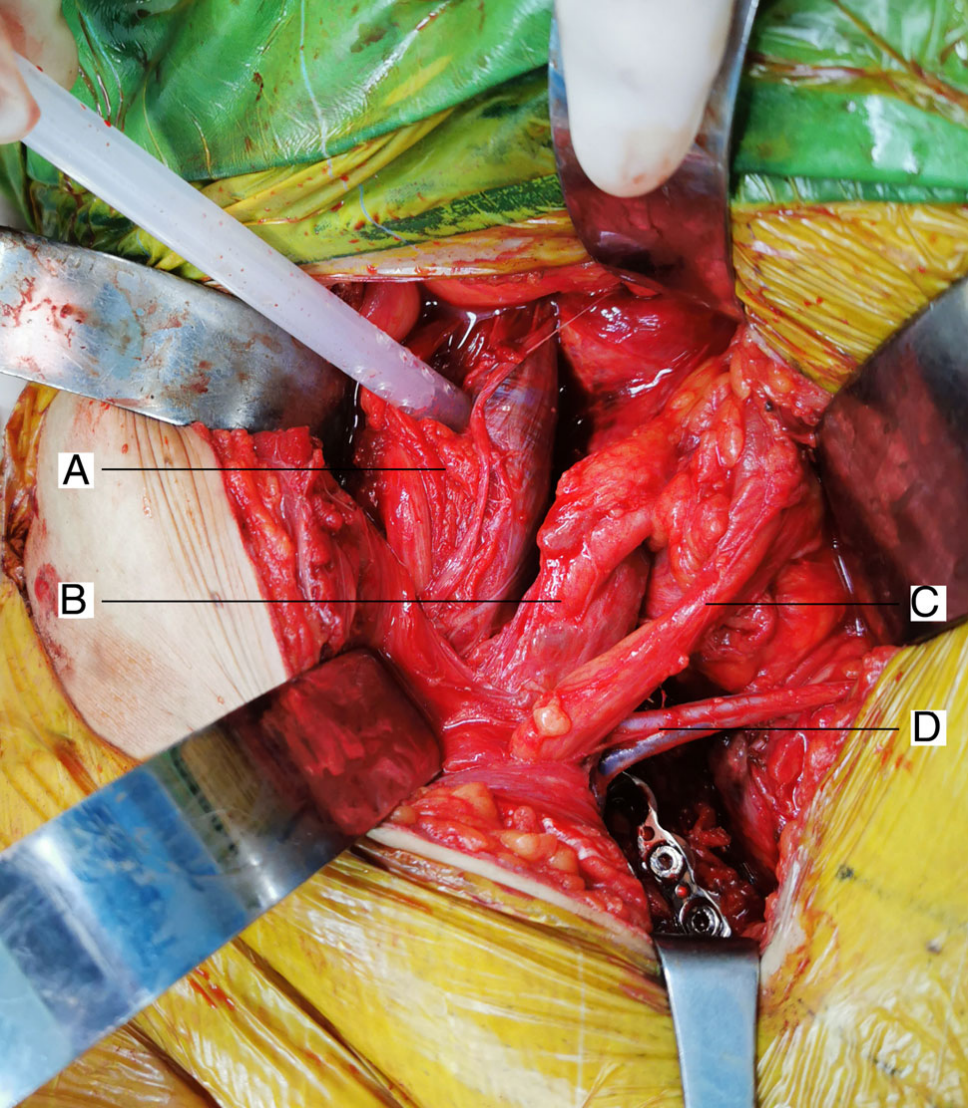
Intraoperative images can clearly reveal important structures.(A) iliac psoas muscle, (B) external iliac artery/vein, (C) spermatic cord, (D) inferior abdominal artery/vein.

Landmarks for the incision were the navel, pubic symphysis and the anterior superior iliac spine (ASIS), as described by professor Keel^4^ (Fig. 1). Along the incision line, the skin, subcutaneous tissue and deep fascia were cut in turn. This was palpated to identify the outer edge of rectus abdominous and cut the external oblique, internal oblique and transverse abdominal muscles along it. We can clearly see important neurovascular structures (Fig. 2). To avoid damaging abdominal wall vessels and the spermatic cord (or round the ligament of the uterus in females), the operation needs to be performed carefully (Fig. 3). Using the “S” hook to pull the peritoneum and pelvic tissues to the inside, and other structures to the outside, to expose the true pelvic ring inside the pelvis. The pararectus surgical approach provides a total of five windows. According to the needs of reduction of the fracture, different windows are selected for exposure. During the operation, the top cone, reduction forceps, Kirschner wires, etc., were used to complete the reduction of the acetabular fracture, the Kirschner wires were used to temporarily fix the fracture, and the reconstruction plate was placed at a suitable location and the screws were screwed in.

#### Ilioinguinal Approach

The incision is made from the anterior 2/3 of the iliac crest along the anterior superior iliac spine and the inguinal ligament to make an arc‐shaped incision which stops at 3 cm above the pubic symphysis. When stripping the attachment point of the iliac muscle and the external oblique tendon, pay attention to avoid damaging the lateral femoral cutaneous nerve until the front of the sacroiliac joint and above the outer ring of the inguinal canal is exposed. After separating the inguinal nerve, spermatic cord or round ligament cut off the rectus abdominis sheath and the joint tendon, pass the traction band from under the inguinal ligament through the femoral nerve bundle and the iliopsoas muscle, and finally incise the iliac pubic fascia to fully expose the fracture end. This approach includes three windows inside, through which three windows are used to expose, reduce and fix fractures in different parts.

#### Statistical Analysis

Use SPSS 23.0 (IBM, Armonk, New York) statistical software to analyze the collected data. Measurement data are expressed as mean ± SD, using a t‐test, counting data using the chi‐squared test. The level of significance was set at *P* < 0.05.

## Results

### Demographic and Clinical Characteristics of Two Approaches

The mean follow‐up period was 19.3 months (range, 12–30 months). The mean age of A approach was 44.5 years (range, 18–69 years),and the mean age of the B approach was 46.5 years (range, 19–71 years). There were 18 male and 12 female patients in group A and 19 male and 11 female patients in group B. According to the Judet and Letournel classification: there was no significant difference (mean ± SD) in fracture classification (*χ* ^2^ = 1.467, *P* > 0.05).

### Clinical Outcomes

The two groups of patients had statistically significant differences in surgical incision length and surgical exposure time (*P* < 0.05). The length of the surgical incision of A approach compared with B approach 11.2 ± 1.5 *vs*. 23.8 ± 2.1 cm (*t* = −26.551, *P* < 0.05) and surgical exposure time 10.8 ± 1.7 *vs*. 19.9 ± 1.9 min (*t* = −19.369, *P* < 0.05). The surgical incision length and surgical exposure time of group A were lower than those of group B. There was no significant difference between the two groups in intraoperative blood loss 591.8 ± 131.4 *vs*. 614.6 ± 132.7 mL (*t* = −0.669, *P* > 0.05). Specific data refer to Table 2.

**TABLE 2:** Comparison of perioperative related indicators between the two groups(^−^x ± s)

Group	(n)	Surgical incision length(cm)	Surgery reveal time(min)	Intraoperative blood loss(ml)
A group	30	11.2 ± 1.5	10.8 ± 1.7	591.8 ± 131.4
B group	30	23.8 ± 2.1	19.9 ± 1.9	614.6 ± 132.7
Statistic	‐	*t* = −26.551	*t* = −19.369	*t* = −0.669
		*P* < 0.05	*P* < 0.05	*P* > 0.05

Group A is the pararectus approach and Group B is the ilioinguinal approach.

### Functional Evaluation and Postoperative Complications

In the A group, 20 patients had excellent results, five patients with good, four patients with fair and one with poor results. There was one patient (3.3%) with postoperative wound fat liquefaction, and the wound completely improved by secretion culture, enhanced dressing and effective antibiotics, one patient (3.3%) developed lateral femoral cutaneous nerve injury. In the B group, 17 patients had excellent results, six patients with good, six patients with fair and one with poor results. One case (3.3%) of postoperative myositis ossificans occurred in the ilioinguinal approach group, and there were no obvious symptoms. There was no significant difference between the two groups in postoperative hip function scores (*χ* ^2^ = 0.417, *P* > 0.05). For specific data refer to Table 3.

**TABLE 3:** Comparison of postoperative hip joint function between two groups (n)

Group	(n)	Improved Merle d' Aubigne and Postel scores			
		Excellent	Good	Fair	Poor
A group	30	20	5	4	1
B group	30	17	6	6	1
Statistic	‐	*χ* ^2^ = 0.417			
		*P* > 0.05			

Group A is the pararectus approach and Group B is the ilioinguinal approach.

## Discussion

Due to the special anatomical position of the acetabulum and its relationship with surrounding tissues, the realization of anatomical reduction of the articular surface has become the goal of treatment for displaced acetabular fractures^7^, ^8^, ^9^. A suitable surgical approach can not only reduce the patient's injury, shorten the operation time, and reduce the patient's bleeding, but also realize the visualization of the fracture, which is beneficial to the reduction and fixation of the fracture. In Letournel's work, the Ilioinguinal approach was introduced in detail. Since this approach can fully expose the anterior wall, anterior column and quadrangular area of the acetabulum, it has always been a classic approach for anterior acetabular fractures^4^, ^10^, ^11^, ^12^. In 2012, Professor Keel^6^ introduced a new surgical approach and named it pararectus. Due to the small incision of this surgical approach, the damage to soft tissues is less, and the front of the acetabulum and the tetragonal area can be observed, which makes this approach in the treatment of anterior acetabular fractures more popular. By directly comparing the pararectus and Ilioinguinal approaches, we found that the former can reduce the length of the surgical incision and shorten the surgical exposure time.

Combining the research situation of the two groups, the authors believe that the pararectus approach has better advantages in the following aspects:(i) fully expose the fracture of the acetabulum, which is conducive to the reduction and fixation of the fracture. Through this incision, we can fully expose from the sacroiliac joint to the anterior pelvic edge of the pubic symphysis, the quadrilateral and the medial part of the posterior column, and the iliac if needed. Mardian *et al*.^13^ reported that in a comparative study of the pararectus approach and the Ilioinguinal approach, the pararectus approach was superior to the Ilioinguinal approach in reducing the gap between the fractures;(ii) during the operation, important blood vessels and nerve structures can be visualized and separated directly. Due to the complicated anatomical relationship of the acetabulum and the abundant nerve and blood vessels, we should always be vigilant when dealing with acetabular fractures. The pararectus approach is converted through five windows, which reduces the iatrogenic damage to nerves and blood vessels^14^, ^15^; (iii) the soft tissue damage is small. Due to the small incision length of the pararectus approach, we fully exposed the fractured and reduced excessive soft tissue damage. Keel *et al*.^16^ reported the pararectus surgical approach for the treatment of 48 cases of acetabular fractures. They mentioned that the surgical approach can provide a clear visualization of the fracture, with an average incision length of 11 cm, which reduces soft tissue damage.

However, the pararectus approach also has shortcomings:(i) the combined acetabular posterior wall fracture cannot be treated with this incision, and the Kocher‐Langenbeck approach is often required, which increases surgical trauma and operation time; (ii)the operation destroys the innervation of the rectus abdominis muscle, which may lead to muscle atrophy, poor healing of the incision and even abdominal wall hernia may occur^17^; (iii) there is a risk of damaging the peritoneum and entering the abdominal cavity. This is mostly related to unfamiliar abdominal anatomy during the operation. Therefore, the surgeon must be familiar with the various anatomical levels. Once the peritoneum is found to be ruptured, it should be sutured in time^18^. At the same time, we should pay attention to the following when using pararectus surgical approach to treat acetabular fractures:(i) for patients who need the Kocher‐Langenbeck approach, adopt the “floating” position before disinfecting the drape, which is conducive to changing to the supine or lateral position during the operation as needed, reducing the operation time; (ii)for patients with severe extraperitoneal adhesions, consider using this surgical approach as appropriate. The Ilioinguinal approach can be used instead; (iii) “crown of death” blood vessels are the anastomotic branch of the inferior abdominal wall arteriovenous or external iliac arteriovenous system and obturator arteriovenous artery and vein^19^, ^20^. This surgical approach can be directly observed on the medial side of the suprapubic branch. Look for the “dead crown” above, and ligate once found to prevent the blood vessel from tearing due to traction during fracture reduction and causing uncontrollable bleeding^21^.

The main limitation of this study is its small size from a single institution. Owing to the relatively short follow‐up time in this study, the mid‐to‐long‐term clinical efficacy requires further follow‐up of patients.

## Authorship Declaration

All authors listed meet the authorship criteria according to the latest guidelines of the International Committee of Medical Journal Editors, and all authors are in agreement with the manuscript.

## References

[1] Panagiotis T , Elias P , Constantinos M , Minos T , Panagiotis D , Elias L . Long‐term results in surgically treated acetabular fractures through the posterior approaches. J Trauma Acute Care Surg, 2007, 62: 378–382. 10.1097/01.ta.0000196540.81630.4e 17297328

[2] Harris AM , Althausen P , Kellam JF , Bosse MJ . Simultaneous anterior and posterior approaches for complex acetabular fractures. J Orthop Trauma, 2008, 22: 494–497. 18670291 10.1097/BOT.0b013e3181830d2a

[3] Wu H , Zhang L , Guo X , Jiang X . Meta‐analysis of modified Stoppa approach and ilioinguinal approach in anterior pelvic ring and acetabular fractures. Medicine, 2020, 99: 183–195. 10.1097/MD.0000000000018395 PMC7004739 31977843

[4] Keel M , Ecker T , Cullmann J , *et al*. The pararectus approach for anterior intrapelvic management of acetabular fractures: an anatomical study and clinical evaluation. J Bone Joint Surg Br, 2012, 94: 405–411. 22371551 10.1302/0301-620X.94B3.27801

[5] Letournel E . The treatment of acetabular fractures through the ilioinguinal approach. Clin Orthop Relat Res, 1993, 292: 62–76. 8519138

[6] Hirvensalo E , Lindahl J , Böstman O . A new approach to the internal fixation of unstable pelvic fractures. Clin Orthop Relat Res, 1993, 297: 28–32. 8242945

[7] Briffa N , Pearce R , Hill A , Bircher M . Outcomes of acetabular fracture fixation with ten years' follow‐up. J Bone Joint Surg Br, 2011, 93: 229–236. 21282764 10.1302/0301-620X.93B2.24056

[8] Daming S , Junhua Z , Wei H . Comparison of the clinical efficacy of pararectus approach and modified Stoppa approach in treatment of acetabular fracture fixation. Orthop Biomech Mater Clin Study, 2019, 16: 38–41.

[9] Tannast M , Najibi S , Matta JM . Two to twenty‐year survivorship of the hip in 810 patients with operatively treated acetabular fractures. J Bone Joint Surg Am, 2012, 94: 1559–1567. 22992846 10.2106/JBJS.K.00444

[10] Matta JM , III Tornetta P . Internal fixation of unstable pelvic ring injuries. Clin Orthop Relat Res, 1996, 329: 129–140. 10.1097/00003086-199608000-00016 8769444

[11] Gänsslen A , Grechenig S , Nerlich M , Müller M , Grechenig W . Standard approaches to the acetabulum part 2: ilioinguinal approach. Acta Chir Orthop Traumatol Cech, 2016, 83: 217–222. 28026721

[12] Fensky F , Lehmann W , Ruecker A , Rueger JM . Ilioinguinal approach: indication and technique. J Orthop Trauma, 2018, 32: 12–13. 10.1097/BOT.0000000000001194 29985894

[13] Märdian S , Schaser K , Hinz P , Wittenberg S , Haas N , Schwabe P . Fixation of acetabular fractures via the ilioinguinal versus pararectus approach: a direct comparison. Bone Joint J, 2015, 97: 1271–1278. 26330596 10.1302/0301-620X.97B9.35403

[14] Keel MJB , Klaus‐Arno S , Moritz T , Bastian JD . The pararectus approach: a new concept. JBJS Essent Surg Tech, 2018, 8: 21–23. 10.2106/JBJS.ST.17.00060 PMC6292723 30588366

[15] Christian vR , Lisa W , Johannes B , *et al*. The pararectus approach for internal fixation of acetabular fractures involving the anterior column: evaluating the functional outcome. Int Orthop, 2019, 43: 1487–1493. 30215099 10.1007/s00264-018-4148-8 PMC6525136

[16] Keel MJB , Tomagra S , Bonel HM , Siebenrock KA , Bastian JD . Clinical results of acetabular fracture management with the pararectus approach. Injury, 2014, 45: 1900–1907. 25457342 10.1016/j.injury.2014.10.040

[17] Bastian JD , Tannast M , Siebenrock K‐A , Keel M . Mid‐term results in relation to age and analysis of predictive factors after fixation of acetabular fractures using the modified Stoppa approach. Injury, 2013, 44: 1793–1798. 24008225 10.1016/j.injury.2013.08.009

[18] Ponsen K‐J , Joosse P , Schigt A , Goslings CJ , Luitse JS . Internal fracture fixation using the Stoppa approach in pelvic ring and acetabular fractures: technical aspects and operative results. J Trauma Acute Care Surg, 2006, 61: 662–667. 10.1097/01.ta.0000219693.95873.24 16967004

[19] Al Talalwah W . A new concept and classification of corona mortis and its clinical significance. Chin J Traumatol, 2016, 19: 251–254. 27780502 10.1016/j.cjtee.2016.06.004 PMC5068213

[20] Kandhari V , Desai M , Bava S , Wade R . Avascular necrosis of acetabulum: the hidden culprit of resistant deep wound infection and failed fixation of fracture acetabulum—a case report. J Orthop Case Rep, 2015, 5: 36. 27299095 10.13107/jocr.2250-0685.341 PMC4845453

[21] Darmanis S , Lewis A , Mansoor A , Bircher M . Corona mortis: an anatomical study with clinical implications in approaches to the pelvis and acetabulum. Clin Anat, 2007, 20: 433–439. 16944498 10.1002/ca.20390

